# 2019 New Year Address of *Zoological Research*

**DOI:** 10.24272/j.issn.2095-8137.2019.001

**Published:** 2019-01-18

At the beginning of this new year full of exciting prospects and potential, we would first like to share our exciting news that *Zoological Research* (*ZR*) is now covered by Science Citation Index Expanded (SCI-E), with full coverage to be backdated to issue 1 in 2016. This encouraging progress demonstrates that *ZR* has taken a critical step on the journey to becoming one of the top journals in the field.

We would like to sincerely thank every author, reader, and colleague of *ZR*. It is your enduring support and faith that has helped in the sustained growth and advancement of *ZR*, which continues to evolve as an important and influential journal. Based on your contributions, the impact of *ZR* has maintained a healthy increasing trend during 2018. In 2017, our Citescore was 0.92 (Elsevier on 31 May 2018), thus ranking *ZR* within the top 20% (161) of the 841 journals listed in the General Medicine category. In addition, the mock impact factor of 2018 (according to current data provided by the Web of Science) has already exceeded that of 2017, up to 1.0.

Our current progress can also be attributed to the great effort made in promoting the journal’s influence. To ensure that the journal provides up-to-date and valuable research findings to academia, we insist on high-quality articles and special issues/collections with support from our esteemed authors and guest editors. Many recent feature articles have attracted considerable attention from peers worldwide, e.g., The role of wildlife (wild birds) in the global transmission of antimicrobial resistance genes (2017, 38n2; Times cited: 13); Tree shrew (*Tupaia belangeri*) as a novel laboratory disease animal model (2017, 38n3; Times cited: 12); Creating animal models, why not use the Chinese tree shrew (*Tupaia belangeri chinensis*)? (2017, 38n3; Times cited: 12); Molecular cloning, pathologically-correlated expression and functional characterization of the colony-stimulating factor 1 receptor (CSF-1R) gene from a teleost, *Plecoglossus altivelis* (2016, 37n2; Times cited: 11); Advances and perspectives in the application of CRISPR/Cas9 in insects (2016, 37n4; Times cited: 9); ZIKA — How fast does this virus mutate? (2016, 37n2; Times cited: 9); and numerous others (data from Web of Science, 9 November 2018).

In 2018, four special issues were successfully released, i.e., “Special Issue for the Animal Model of Infectious Diseases” (39n1; guest editor: Yu-Hai Bi, Institute of Microbiology, Chinese Academy of Sciences (CAS), China), “Herpetofaunal Diversity in Indochina” (39n3; guest editors: Nikolay A. Poyarkov, Jr., Lomonosov Moscow State University, Russia; Jing Che, Kunming Institute of Zoology, CAS, China); “Special Issue for Primates and Primatology in China” (39n4; guest editor: Pengfei Fan, Sun Yat-Sen University, China); “Mammal Diversity in Asia” (39n5; guest editors: Kai He, Kunming Institute of Zoology, CAS, China; Masaharu Motokawa, Kyoto University, Japan; Xue-Long Jiang, Kunming Institute of Zoology, CAS, China), comprising authors from the US, Canada, Russia, Belgium, Cambodia, Brazil, Thailand, and others.

*ZR* has also maintained active and regular participation in both academic and publication conferences, e.g., 1^st^ AsiaEvo Conference, 8^th^ International Symposium on Primate Research, Annual Conference of ScholarOne Users, and Development Forum of Science, Technical and Medical (STM) Journals of China. After successfully convening the Frontiers in Zoology Symposium for the last two years, *ZR* will announce the next symposium in February 2019 with the theme of “Protection and Utilization of Animal Resources”. We hope to see many in attendance at the conference, with all relevant information to be found on the *ZR* homepage.

Importantly, *ZR*’s editorial board members and generous readers and authors have all performed their duties impeccably and promoted the journal within academic institutes and to colleagues both inside and outside of China. Our efforts have paid off, with more than 60% of yearly peer reviewers now located outside of China, and the database of reviewers, authors, readers, homepage visitors, and WeChat followers seeing significant expansion. We would like to thank all the reviewers and editors for their patience and contributions, which have greatly helped in maintaining the high-quality manuscripts published in *ZR*.

Last but not the least, *ZR* has had nine new editorial boards by the end of 2018. We are sincerely grateful for all the hard work of each editorial board member in guaranteeing the scientific value of the journal and maintaining its positive roles in academia. The 10^th^ editorial board will commence at the start of 2019 and includes 53 outstanding scientists from 14 countries. To ensure a vibrant and effective team, we encourage scholars with passion and ideas for cultivating *ZR* into a more influential journal to join the team throughout the year. If you are interested in working with our team, please do not hesitate to contact us.

Scientific journals are of considerable value and strategic significance. The Chinese government recently emphasized that the development of scientific journals is critical for advancing innovation in China, increasing the academic influence of Chinese scientists among international peers, strengthening the nation’s discursive power in promoting international scientific and cultural communications, and boosting the transformation and development of the scientific publication industry in China. Ensuring the continuing vibrancy and influence of Chinese scientific journals is not only the expectation of generations of Chinese scientists and publishers, but is also crucial for propelling scientific and technological advancement. Among the 5 052 (as of 31 December 2017) scientific/technology/medical journals of China, *ZR* is still young; however, it has shown great vitality, considerable potential, and a clear outlook in advancing the scientific spirit.

It is an exciting time for *ZR* as it grows, whilst remaining adaptable, motivated, and open to new ideas. *ZR* will continue to focus on publishing exciting results on: 1) Primates and Animal Models; 2) Conservation and Utilization of Animal Resources; and 3) Animal Diversity and Evolution. We further hope for your enduring involvement into the future. *ZR* is prepared to be challenged, stimulated, and inspired. Again, thank you all! May your new year hold great promise and many good wishes!

Sincerely


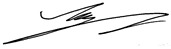

Yun Zhang, Executive Editor-in-Chief *Kunming Institute of Zoology*, *Chinese Academy of Sciences, Kunming Yunnan 650223, China* 
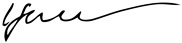

Yong-Gang Yao, Editor-in-Chief *Kunming Institute of Zoology*, *Chinese Academy of Sciences, Kunming Yunnan 650223, China*
